# Disability and Comorbidity: Diagnoses and Symptoms Associated with Disability in a Clinical Population with Panic Disorder

**DOI:** 10.1155/2014/619727

**Published:** 2014-03-02

**Authors:** Caroline A. Bonham, Eberhard Uhlenhuth

**Affiliations:** ^1^Department of Psychiatry, Center for Rural and Community Behavioral Health, University of New Mexico, MSC09 5030, Albuquerque, NM 87131, USA; ^2^Department of Psychiatry, University of New Mexico, MSC09 5030, Albuquerque, NM 87131, USA

## Abstract

*Background*. Anxiety disorders are associated with considerable disability in the domains of (1) work, (2) social, and (3) family and home interactions. Psychiatric comorbidity is also known to be associated with disability. *Methods*. Data from the Cross-National Collaborative Panic Study was used to identify rates of comorbid diagnoses, anxiety and depression symptom ratings, and Sheehan disability scale ratings from a clinical sample of 1165 adults with panic disorder. *Results*. Comorbid diagnoses of agoraphobia, major depression, and social phobia were associated with disability across the three domains of work, social, and family and home interactions. The symptom of agoraphobic avoidance makes the largest contribution to disability but there is no single symptom cluster that entirely predicts impairment and disability. *Limitations*. The findings about the relative contributions that comorbid diagnoses make to disability only apply to a population with panic disorder. *Conclusions*. Although panic disorder is not generally considered to be among the serious and persistent mental illnesses, when it is comorbid with other diagnoses, it is associated with considerable impairment. In particular, the presence of agoraphobic avoidance should alert the clinician to the likelihood of important functional impairment. When measuring the functional impact of comorbid anxiety disorders, both the categorical and the dimensional approaches to diagnosis make valuable contributions.

## 1. Background

Epidemiological studies have demonstrated that panic disorder is associated with significant morbidity and impairment [1]. Community samples have also confirmed that agoraphobia, among the different phobias, is particularly associated with impairment. Panic disorder with agoraphobia is associated with increased self reports of disability compared to either agoraphobia or panic disorder alone [2]. Agoraphobia is also associated with higher rates of help seeking and medication use compared to simple phobia and social phobia [3].

Several epidemiological studies also confirmed that comorbidity is associated with impairment. In the National Comorbidity Survey, those with comorbid anxiety or phobic disorders were more symptomatic than those with pure disorders [3] as measured by perceived role impairment, help seeking, and medication use. Interestingly, this study also found that perceived role impairment was less prevalent amongst those with agoraphobia than those with simple or social phobia; however, this difference disappeared amongst those who also have panic attacks. Amongst patients with anxiety disorders, the presence of comorbid depression indicated a more chronic course with worse prognosis [4]. The National Comorbidity Survey also clearly demonstrated that panic disorder is highly comorbid with other phobic and depressive diagnoses and that panic disorder with agoraphobia is especially associated with increased comorbidity [5].

In a prospective study with a large clinical sample, Bruce et al. showed that comorbidity is a marker of impairment in anxiety disorders and that adults with more than one comorbid anxiety diagnosis were less likely to recover and more likely to have a recurrence than adults with a single anxiety diagnosis [6]. And a smaller clinical study with 61 patients demonstrated that the presence of depression is a risk factor for worse quality of life amongst patients with panic disorder [7].

Epidemiological studies have also confirmed that a diagnosis of major depressive disorder is also associated with considerable role impairment [8]. And it is recognized that there is an overlap in the symptoms of depression and anxiety [9] since as many as 95% of patients with major depressive disorder report symptoms of anxiety [10].

Although it is well known that phobic and depressive diagnoses are associated with impairment and that there are high rates of comorbidity between these disorders, it is not known whether particular phobic and depressive disorders make independent contributions to the total burden of disability. Also, there is little known about which symptoms of phobias or depression contribute to overall disability. The sample of the Cross-National Collaborative Panic Study provides an opportunity to confirm previous findings within a large and extensively studied clinical sample. Strengths of this study include its large sample size and the use of psychiatric diagnoses made by clinicians using a structured interview. In addition, this sample presents the opportunity to investigate whether particular diagnoses make independent contributions and whether different symptoms of phobic and depressive disorders contribute separately to overall disability.

## 2. Aims

The aims of this study are as follows:

1. to confirm previous findings about predictors of impairment in a large clinical sample,
2. to identify the patterns of disability associated with different comorbid (depressive and phobic) diagnoses in a population of patients with panic disorder,
3. to identify which symptoms of these comorbid diagnoses account for these patterns of disability.

## 3. Methods

### 3.1. Patients

Patients aged 18–65 who met DSM-IIIR criteria for lifetime diagnosis of panic disorder with or without agoraphobia were included in the Cross-National Collaborative Panic Study—part 2—if they had at least one panic attack a week in the three-week period just prior to assessment. Patients were excluded if they had a diagnosis of obsessive compulsive disorder, bipolar disorder, any psychosis, substance abuse or dependence, or major depressive disorder with onset prior to panic disorder, that is, “primary major depressive disorder.” Current diagnoses of comorbid conditions were made by a clinician at baseline prior to entry into the study using a modified version of the SCID based on DSM III-R diagnostic criteria (SCID-UP) [11]. According to the SCID-UP, PDAG was categorized as either panic disorder with limited phobic avoidance or as panic disorder with extensive phobic avoidance. For the purposes of this analysis, only those with extensive phobic avoidance were considered to have PDAG. Patients were divided into groups depending on the presence of a current comorbid phobic or depressive disorder diagnosis. Additionally, it is important to note that, in the SCID-UP, the term “simple phobia” was used rather than the current term “specific phobia.” For the sake of accuracy, we have elected to use the term “simple phobia” when referring to diagnoses made before 1996 when the term was changed with the publication of DSM-IV. Similarly, we will use the term “social phobia” rather than “social anxiety disorder.”

The initial study included 1168 patients with panic disorder. We excluded three patients because they did not meet criteria for panic disorder based on the number of panic attacks reported at study entry. Please see references for a full description of the study [12, 13].

### 3.2. Instruments

At baseline, each participant completed the 21-item Hamilton Rating Scale for Depression (HRSD) [14], the Hamilton Rating Scale for Anxiety (HRSA) [15], and the Sheehan Disability Scale which includes subscores of disability in the domains of family and home, social function, and work [16].

The extent of agoraphobia, the extent of the worry about panic attacks, and the intensity of panic attacks were each rated from 0 to 10 with 10 being the worst. Each respondent also reported the number of “situational” or “cued” panic attacks and the number of spontaneous panic attacks experienced in the preceding week. The participants kept a diary of their panic attacks and received training on the identification of panic attacks and their subtypes. Independent consultants reviewed the panic attack diaries as part of the quality assurance program.

The extent of comorbidity of these diagnoses was determined by determining if each individual filled the criteria for any of the following five diagnoses: agoraphobia, social phobia, specific phobia, major depressive disorder, or dysthymia. Less than 10% of the sample had more than three comorbid diagnoses; therefore the sample was divided into four groups: (1) those with uncomplicated panic disorder without comorbidity, (2) those with panic disorder and one comorbid diagnosis, (3) those with panic disorder and two comorbid diagnoses, and (4) those with panic disorder and more than three comorbid diagnoses.

### 3.3. Analyses

Mean Sheehan Disability Scale scores for patients with a single comorbid diagnosis were compared by *t*-test to those with panic disorder without comorbidity. Multiple regression was used to determine the independent contribution of each comorbid diagnosis and gender. There were no significant interactions between gender and any of the comorbid diagnoses so that the multiple regressions were repeated with main effects of the comorbid diagnoses only. Spearman's test was performed to evaluate the correlation between each of the anxiety and phobic symptom ratings and disability as measured by the Sheehan Disability Scale.

A second multiple regression model was constructed to determine the independent contribution of each anxiety and depressive symptom to the Sheehan disability score. The Hamilton Anxiety Score, the Hamilton Depression Score, and ratings of simple phobia symptoms, social phobia symptoms, agoraphobia symptoms, and number of situational and spontaneous panic attacks in the preceding week were entered as independent variables.

Given the large size of this sample, we adopted a probability of *P* ≤ 0.01 as the criterion of statistical significance.

## 4. Results

### 4.1. Demographics

61% of the sample was female and 39% was male. The mean age was 34. Comorbid anxiety and depressive disorders were common and included agoraphobia (35.8%, *N* = 417), social phobia (15%, *N* = 176), simple phobia (29%, *N* = 335), current major depressive disorder (16%, *N* = 176), and dysthymia (11%, *N* = 130). Thirty-three percent of the sample had uncomplicated panic disorder (*N* = 369); 39% had one comorbid diagnosis (*N* = 438); 19% had two comorbid diagnoses (*N* = 219); and 9.7% had three or more comorbid diagnoses (*N* = 110).

### 4.2. Findings

**Figure 1:**
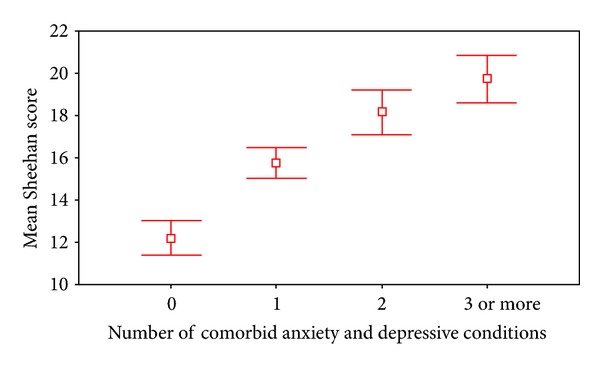
Number of comorbid conditions by mean total Sheehan score.

Table 1 shows that among patients with panic disorder, comorbid diagnoses of agoraphobia, major depression, and, to a lesser degree, social phobia were associated with increased disability in all three domains of home, work, and social function. Interestingly, comorbid dysthymia or social phobia was not associated with increased disability compared to the subsample with pure panic disorder.  Table 2 shows the result of the linear regression equation and demonstrates that comorbid agoraphobia and major depressive (and social phobia to a minor degree) each made independent contributions to the level of increased disability. Using Cohen's definitions [17], agoraphobia's effect on overall disability was a medium sized effect and this appeared to be due mainly to agoraphobia's effect on the social domain of disability. All other significant effect sizes were small.  Table 3 shows that increased disability scores were correlated with higher levels of most phobic and depressive symptoms as well as the number of panic attacks.  Table 4 shows the relative independent contributions of the different phobic and depressive symptoms to the total disability scores. Being consistent with the findings shown in Table 3, all of the anxiety and depressive symptoms make independent contributions to disability with the striking exception of the number of spontaneous panic attacks. Across all domains, agoraphobia's effect on disability was consistently the largest effect identified.  Figure 1 demonstrates that increased comorbidity was associated with higher disability scores.

## 5. Discussion

With respect to the first aim of our study, this analysis of a large, clinical sample of individuals with panic disorder confirmed that the presence of comorbid agoraphobia, major depressive disorder, or social phobia is associated with increased levels of impairment. Of these three comorbid diagnoses, agoraphobia appears to confer the largest degree of additional impairment.

Our second aim was to identify patterns of disability associated with these comorbid diagnoses. Agoraphobia and major depressive disorder are associated with disability across all the three domains: family, social, and work. Social phobia appears to contribute to increased levels of disability primarily through its effect on the social domain.

Thirdly, when exploring the symptoms of anxiety and depression and the associated patterns of disability, we found that the number of situational panic attacks, the degree of worry associated with panic attacks, and the intensity of panic attacks were all associated with disability primarily through their effect on the social domain. Interestingly, elevated scores on the HRSD were associated with increased disability in the domains of family and work but the association between HRSD scores and disability in the social domain did not reach statistical significance. Finally, agoraphobia and the total score on the HRSA were associated with disability across all three realms of disability.

Increased rates of agoraphobic avoidance, worry, panic attack intensity, number of situation panic attacks, and higher scores on HRSA and HRSD were all associated with disability; yet it was the quantitative rating of agoraphobic avoidance that was most highly associated with disability.

Previous work also has identified agoraphobia as an indicator of poor prognosis. A large prospective study of a clinical sample of patients with generalized anxiety disorder, social phobia, and panic disorder found that the presence of agoraphobic avoidance increased recurrence and decreased chance of recovery of these anxiety disorders [6]. In a report following up on 423 subjects from the Cross-National Collaborative Panic Study, the presence of agoraphobia at baseline predicted impairment at followup after 4 years [18]. In a clinical sample of 125 patients with panic disorder, the presence of agoraphobic avoidance predicted worse quality of life [19].

In contrast, a study of a Norwegian community sample that looked at quality of life in different anxiety disorders concluded that agoraphobia “does not seem to imply a serious disorder.” The questions in this study were written to capture quality and meaning to life rather than disability and they found that social phobia had the strongest effect whereas the presence of agoraphobia was negligible [20].

In our study, when looking at individual symptoms, all hypothesized symptoms contributed to disability except for spontaneous panic attacks. This finding is consistent with other work. MacAndrew and colleagues [21] found that situational panic attacks were associated with impairment of functioning even if panic disorder was not present. Spontaneous panic attacks establish the diagnosis of panic disorder; however, situational panic attacks are probably more related to phobic symptoms. It has also been shown that, in contrast to spontaneous panic attacks, situational panic attacks are more closely related to the presence of cognitive distortions as well as the presence of agoraphobia [22].

Kennedy et al. [23] compared the Sheehan disability scores among patients with anxiety and depression. They found that those with MDD or mixed anxiety disorders had significantly higher disability in the domain of family than those with social phobia or panic disorder alone. There was no significant difference between the diagnoses in the realms of social or work impairment. They also found that social phobia was mostly associated with impairment in the social domain rather than in the work and family domains. These findings are consistent with ours.

According to an analysis of the National Comorbidity Survey data, the probability of seeking help and using medication is higher at a given level of perceived role impairment for those with agoraphobia in contrast to those with simple or social phobia. However, a smaller proportion of those with agoraphobia were likely to report role impairment compared to those with other phobias. And there was no significant difference in perceived role impairment by type of phobia in those with panic attacks. This study also showed that specific phobia is associated with functional impairment; however, it was not clear whether the addition of specific phobia causes any further impairment when it is comorbid with another diagnosis [3]. With regard to this last point, our clinical study suggests that specific phobia does not confer any additional disability when panic disorder is present.

In a large community sample, Stinson et al. found that the presence of specific phobia was associated with considerable impairment as measured by the Mental Component Summary of the SF-12, v2. Using this measure, Stinson et al. demonstrated that the contribution of specific phobia to disability was similar to that of other anxiety disorders [24]. These findings were in contrast to those from a community sample in New Zealand which found that specific phobia was associated with less impairment than other anxiety disorders [25]. This latter study used the Sheehan Disability Scale as part of its assessment of disability which may explain the congruence with our findings.

In our study, there was no single explanatory concept to predict disability in this population. To some extent, the number of comorbid diagnoses predicted disability. However, the presence of particular diagnoses and symptoms also predicted disability. The presence of agoraphobic avoidance had the largest effect on disability of the symptoms considered. Similarly, of the various diagnoses examined, comorbid agoraphobia was most highly associated with increased disability.

## 6. Limitations

In this sample, there is no comparison group without a panic disorder diagnosis and therefore findings cannot be generalized to anxiety or depressive diagnoses that are not comorbid with panic disorder. Primary major depressive disorder and bipolar disorder were excluded from this sample and therefore we are unable to draw any conclusions about major depressive disorder or bipolar disorder that preceded the diagnosis of panic disorder despite the high comorbidity of these conditions. Findings from the National Comorbidity Survey indicate that approximately 48% of those with both panic disorder and major depressive disorder had the onset of their depression prior to the development of panic disorder [26]. However, in Roy-Byrne et al.'s analysis of the clinical features of comorbid panic disorder and depression, controlling for the temporal sequence of panic disorder did not alter the outcome [27]. Additionally, information about the presence of personality disorders was not collected as part of this sample. Finally, with 13 sites, diagnoses of comorbid diagnoses may not have been consistent despite training and monitoring.

## 7. Conclusion

Our findings suggest that comorbid diagnoses of agoraphobia, social phobia, and major depression make independent contributions to disability, with agoraphobia having the largest effect of these three. Awareness of these patterns of disability may be helpful for clinicians when identifying therapeutic goals. Clinically, it is clearly important to be attentive to the presence of agoraphobic avoidance. Due to the consistent association of agoraphobia with functional impairment, we would echo the suggestions in the APA practice guideline that combined CBT and antipanic medication should be considered in patients with panic disorder and severe agoraphobia [28].

This study also has implications for the ongoing discussion as to whether anxiety disorders should be classified dimensionally or categorically [29]. The findings from this study suggest that both methods of classification have value when considering the functional impact of these disorders. The finding that different comorbid diagnoses make separate independent contributions to disability supports the categorical approach to diagnosis. However, the dimensional approach to diagnosis is supported by our finding that the total number of comorbid diagnoses has a cumulative effect.

Lastly, this study confirms that, although panic disorder is not generally considered to be among the serious and persistent mental illnesses, it is clear that, when it is comorbid with other diagnoses, panic disorder is associated with considerable functional impairment.

## Figures and Tables

**Table 1:** *t*-test results of comorbid conditions and disability.

Comorbid diagnosis	*n*	Sheehan total score			Family domain			Social domain			Work domain		
		Mean	SD	*d*	Mean	SD	*d*	Mean	SD	*d*	Mean	SD	*d*
Agoraphobia													
Absent	333	12.2	7.09	0.78	3.43	2.78	0.51	4.45	2.94	0.97	4.45	3.03	0.47
Present	179	17.93**	6.78		4.99**	3.20		7.27**	2.44		6.09**	3.31	
Social phobia													
Absent	333	12.2	7.09	0.22	3.43	2.78	0.08	4.45	2.94	0.3	4.55	3.03	0.09
Present	43	14.6	7.35		3.79	3.20		5.84**	3.08		4.98	3.07	
Simple phobia													
Absent	333	12.2	7.09	0.07	3.43	2.78	0.01	4.45	2.94	0.11	4.55	3.03	0.06
Present	110	12.77	6.87		3.45	3.00		4.85	3.17		4.32	2.86	
MDE													
Absent	333	12.2	7.09	0.46	3.43	2.78	0.44	4.45	2.94	0.27	4.55	3.03	0.31
Present	40	17.31**	6.8		5.38**	2.60		5.73**	2.74		6.02**	2.85	
Dysthymia													
Absent	333	12.2	7.09	0.15	3.43	2.78	0.07	4.45	2.94	0.22	4.55	3.03	0.05
Present	35	14.06	7.93		3.75	2.97		5.54	3.09		4.79	3.33	
Gender													
Male	452	15.24	7.48	0.07	4.10	2.96	0.15	5.56	2.96	0.11	5.54	3.00	0.09
Female	710	15.75	7.52		4.56	3.23		5.88	3.09		5.26	3.41	
Marital status													
Married	632	15.13	7.59	0.15	4.49	3.13	0.05	5.56	3.13	0.26	5.23	3.21	0.12
Single	370	16.26	7.33		4.31	2.96		6.39**	2.87		5.65	3.25	
Employment status													
Employed	634	14.37	7.78	0.41	3.98	2.98	0.35	5.57	2.99	0.23	4.85	3.09	0.44
Unemployed	369	17.52**	7.09		5.06**	3.10		6.27**	3.11		6.29**	3.26	

**P < 0.001, *P < 0.01 by *t*-test; *d*: Cohen's *d*; MDE refers to major depressive episode.

**Table 2:** Linear regression of comorbid conditions and disability.

Comorbid diagnosis	*n*	Sheehan total score		Family domain		Social domain		Work domain	
		*b*	Partial *r* ^2^	*b*	Partial *r* ^2^	*b*	Partial *r* ^2^	*b*	Partial *r* ^2^
Agoraphobia	417	4.79**	0.31	1.37**	0.22	2.23**	0.36	1.23**	0.18
Social phobia	176	1.63*	0.08	0.38	0.05	0.91**	0.11	0.34	0.04
Simple phobia	335	0.08	0.01	0.04	0.01	−0.06	−0.01	−0.19	−0.03
MDE	181	3.44**	0.17	1.26**	0.15	0.70*	0.08	1.34**	0.15
Dysthymia	130	0.69	0.03	0.52	0.06	0	0.00	0.05	0.01

Overall model (*R* ^2^)			0.19**		0.12**		0.19**		0.12**

**P < 0.001, *P < 0.01; MDE refers to major depressive episode.

Adjusted for age, marital status and employment status.

**Table 3:** Spearman correlation values between symptoms and disability scores.

Symptoms	Sheehan total score	Family domain	Social domain	Work domain
Agoraphobia	0.50**	0.36**	0.47**	0.36**
Situational panic attacks	0.27**	0.21**	0.29**	0.19**
Spontaneous panic attacks	0.11*	0.12**	0.06	0.13**
Worry	0.30**	0.24**	0.31**	0.26**
Intensity	0.32**	0.22**	0.32**	0.23**
HRSA	0.43**	0.44**	0.40**	0.42**
HRSD	0.41**	0.39**	0.35**	0.40**

**P < 0.001, *P < 0.01; HRSA refers to Hamilton rating scale for anxiety; HRSD refers to Hamilton rating scale for depression.

**Table 4:** Linear regression of disability and anxiety and depression symptoms.

Symptoms	Sheehan total score		Family domain		Social domain		Work domain	
	*b*	Partial *r* ^2^	*b*	Partial *r* ^2^	*b*	Partial *r* ^2^	*b*	Partial *r* ^2^
Agoraphobia	0.76**	0.30	0.19**	0.17	0.29**	0.27	0.20**	0.17
Situational panic attacks	0.14*	0.07	0.04	0.05	0.08**	0.10	0.02	0.03
Spontaneous panic attacks	0.00	0.01	0.00	0.01	0.00	0.00	0.00	0.01
Worry	0.02*	0.09	0.01	0.08	0.01	0.10	0.01	0.05
Intensity	0.29*	0.10	0.07	0.06	0.15**	0.13	0.08	0.06
HRSA	0.12*	0.13	0.08**	0.21	0.06**	0.16	0.06*	0.15
HRSD	0.20**	0.18	0.07**	0.16	0.05	0.11	0.10**	0.21

Overall model (*R* ^2^)		0.36**		0.26**		0.32**		0.24**

**P < 0.001, *P < 0.01; HRSA refers to Hamilton rating scale for anxiety; HRSD refers to Hamilton rating scale for depression.

Adjusted for age, marital status, and employment.
